# The Effects of Chronic Opioid Therapy on Achalasia and the Upper Esophageal Sphincter

**DOI:** 10.3390/medsci13030150

**Published:** 2025-08-22

**Authors:** Joshua Kalapala, Promila Banerjee, Emma Schnittka, Christine Son, Jeff Leya, Stephen Sontag, Thomas Schnell, Bani Chander-Roland

**Affiliations:** 1 Loyola Stritch School of Medicine, MercyOne Hospital, North Iowa 2160 S 1st Ave, Maywood, IL 60153, USA; promila.banerjee@va.gov; 2 Edward Hines VA Hospital, 5000 5th Ave, Hines, IL 60141, USA; jeff.leya@meryone.org (J.L.); stephen.sontag@va.gov (S.S.); thomas.schnell@va.gov (T.S.); 3 Loyola Medical Center, 2160 S 1st Ave, Maywood, IL 60153, USA; emma.schnittka@luhs.org (E.S.); christine.son@luhs.org (C.S.); 4 Brooklyn VA Hospital, 800 Poly PI, Brooklyn, NY 11209, USA; bani.chander-roland@va.gov

**Keywords:** upper esophageal sphincter, opioid, achalasia, esophageal dysmotility, dysphagia

## Abstract

**Background:** The rise of opioid drug usage in the U.S. correlates with increasing recognition of gastrointestinal side effects, especially in the esophagus. The literature has recently noted that abnormalities in the upper esophageal sphincter (UES) are a poor prognostic factor in Achalasia treatment response. A better understanding of the relationship between opioid therapy and esophageal motility and sphincter function may shape our management guidelines for esophageal dysmotilities. This study aimed to evaluate dysmotility patterns, specifically UES function, among the veteran population, where opioid use is reportedly high. **Methods:** We performed a retrospective search of all the veterans at a large urban veteran affairs hospital who had undergone esophageal manometry from 2013 to 2022, collecting data on patient demographics, indication for procedure, diagnosis, sphincter pressure values, and presence of chronic opioid use. **Results:** Of 395 patients, 29% had a history of chronic opioid therapy. Notably, patients that were diagnosed with Achalasia had a greater proportion of chronic opioid use as compared to those who were not. Additionally, there was a statistically significant lower average upper esophageal resting pressure in opioid patients compared to non-opioid patients. **Conclusions:** Veteran patients with Achalasia have a greater proportion of chronic opioid use as compared to those without. There are significant manometric pressure differences at the upper esophageal sphincter among chronic opioid users when compared to non-opioid users.

## 1. Introduction

The dramatic rise in the use of opioid drugs in the United States correlates with the increasing recognition of various types of physiological gastrointestinal side effects, specifically affecting the motility of the esophagus and gastrointestinal tract. Recently published studies have reported an increased frequency of esophagogastric junction outflow obstruction (EGJOO), Achalasia, and lower esophageal sphincter (LES) dysfunction [1,2,3,4,5] among opioid users. Additionally, motility disorders such as Achalasia are increasing in incidence and prevalence [6]. Similarly, other studies in the literature have found a relationship between opioid use and smooth muscle sphincters. For instance, one study noted that morphine causes a spastic effect at the sphincter of Oddi, while an animal trial observed a dyssynergia of the external urethral sphincter in cats due to agonism of k-2 opioid receptors [7,8,9].

To date, no studies have assessed the relationship of upper esophageal sphincter (UES) motility dynamics among chronic opioid users. Furthermore, this has never been studied among veterans who reportedly have a higher prevalence of opioid use than the general patient population [10,11]. The UES is noteworthy of study as the literature has further noted that its pathophysiology has significant implications on overall GI health. For instance, UES abnormalities in patients with concurrent achalasia are found to have poor treatment response as compared to those with normal UES function [12,13]. Therefore, a better understanding of the relationship between opioid therapy and esophageal motility and sphincter function is especially important in the Western world and in veterans, where opioid use is on the rise. Furthermore, this may guide some approaches into opioid vs. non-opioid pain management strategies in patients with pre-existing esophageal dysmotility.

The main objectives of the present study are to evaluate esophageal dysmotility patterns, including the esophageal sphincter (UES and LES) functions, as well as manometry indications, and risk factors among chronic daily opioid use in the veteran patient population.

## 2. Materials and Methods

We conducted a retrospective database search using the Computerized Patient Record System (CPRS) at Edward Hines VA Hospital, including all Hines veterans who had undergone the esophageal manometry procedure between 1997 and 2022. For procedures between 1997 and 2012, we collected data from the Water Perfusion Manometry System and for those between 2013 and 2022, we used the High-Resolution Manometry (HRM) Medtronic Manoview ESO 3.3 software. Given the heterogenicity of the two different modalities, we homogenized the data set by only utilizing the HRM data from 2013 to 2022. We excluded patients with missing manometry data, those with esophageal cancers, and those with post-op strictures.

Chronic opioid use was defined as continuous daily use for 90 days [14] prior to and including the manometric procedure. This was collected by reviewing the Patient Pharmacy Records.

Group Assignment: Major variables were examined, and patients were assigned to both the indication group, according to the indication stated on the consultation request, and the manometric finding group, according to the manometry report.

THE INDICATION GROUP COMPRISED THE FOLLOWING: Pulmonary Symptoms (PUL), Chest Pain (CP), Known Achalasia (ACH), GERD (GER), Dysphagia (DYS), and Post-op Dysphagia (POD).

THE MANOMETRIC GROUP COMPRISED THE FOLLOWING MOTILITY FINDINGS: UES Hypertension (IR), UES Hypotension (UES Hypo), Achalasia (ACH), Esophagogastric Outflow Obstruction (EGJOO), Distal Esophageal Spasm (DES), Ineffective Esophageal Motility (IEM), Hypercontractile Esophagus (Jack-Nut), LES Hypotension (IEM), and Normal.

These diagnoses were categorized into three groups:

Normal LES/Body (Normal Manometry Study).

Achalasia.

Non-Achalasia Dysmotilities.

THE PRESSURE ANALYSIS GROUPS COMPRISED THE FOLLOWING MOTILITY FINDINGS: UES Resting Pressure, UES Residual Pressure, LES Resting Pressure, LES Residual Pressure/IRP, and DCI.

Statistical Methods:

The Fischer Exact Test for two-variable comparisons was used to assess the following:

The frequency of categorical variables (opioid usage) among patients in the Achalasia and Non-Achalasia diagnostic groups.

The Chi Square tests of significance were used to compare the following:

Demographic data (Gender Distribution) between the opioid and non-opioid groups.

The frequency of categorical variables (opioid usage) among average LES and UES pressures.

The frequency of categorical variables (opioid usage) among all indication groups

Student’s *t* test was used to compare the average values of continuous variables among opioid users and non-users.

## 3. Results

A total of 395 patients were studied (343 male, 52 female) (mean age at time of procedure: 69; range 31–96, median: 66). The patient demographics stratified by opioid use and no opioid use are listed in Table 1. The demographics of both groups are statistically similar as noted by the *p*-values.

Of these patients, 111 (~29%) had a history of chronic opioid therapy. One patient who had aborted esophageal manometry (ESM) was excluded.

**Figure 1:**
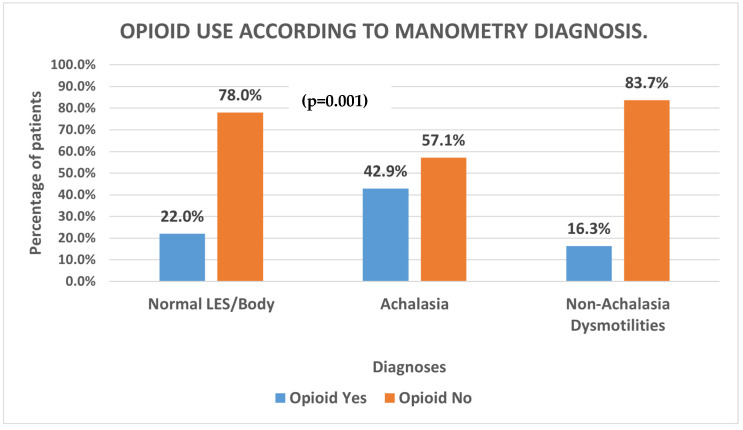
Opioid use in each manometry diagnostic group.

In our comparison of esophageal motility diagnoses, we found that patients diagnosed with Achalasia had a statistically higher proportion of chronic opioid use as compared to those with Non-Achalasia dysmotilities on manometry (Figure 1; *p* = 0.001).

In our analysis of the Non-Achalasia dysmotilities, we did not observe a statistically high prevalence of opioid users having EGJOO compared to other esophageal dysmotilities (*p* = 0.07); however, there was a trend toward it.

**Figure 2:**
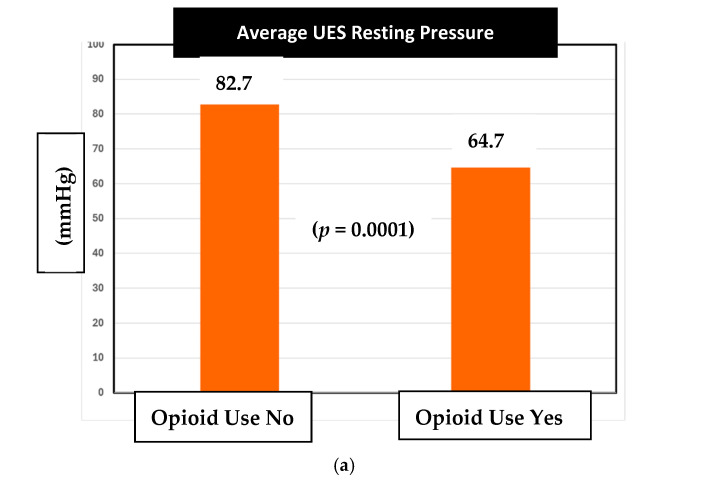

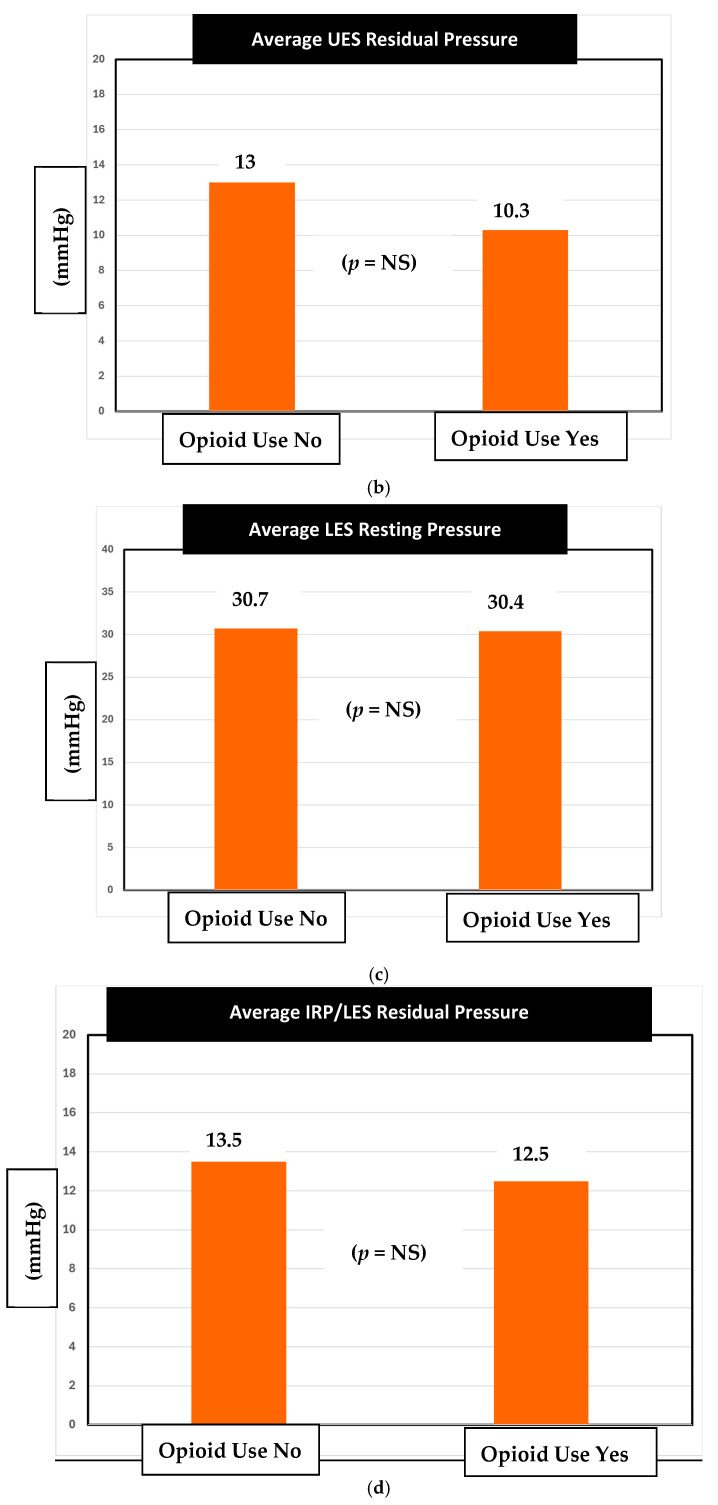
(**a**) Effect of opioid use on UES resting pressure; (**b**) effect of opioid use on UES residual pressure; (**c**) effect of opioid use on LES resting pressure; (**d**) effect of opioid use on IRP/LES residual pressure.

Next, in our assessment of sphincter changes associated with chronic opioid use, there was a statistically significant difference observed in the average UES resting pressures between opioid and non-opioid users (Figure 2a). Specifically, the mean UES resting pressure in opioid users was 64.7 mm Hg compared to the 82.7 mm Hg pressure in non-opioid users (***p*** = **0.0001**).

The normal range of UES resting pressure in Medtronic Manoview ESO 3.3 software is 34–104 mmHg. Therefore, both opioid and non-opioid patients had their average UES resting pressures within the normal range.

Alternatively, there was no statistically significant difference observed between the UES residual pressure values between the opioid and non-opioid groups (Figure 2b). We found that opioid users had an average UES residual of 10.3 mmHg, while the non-opioid users had an average of 13 mmHg (*p* = 0.06).

In our analysis of LES resting pressure, there was no difference in average LES resting pressure between non-opioid users and opioid users (Figure 2c). The average pressure of non-opioid users was 30.7 mmHg while the average pressure for opioid users was 30.4 (*p* = 0.89). According to Medtronic Manoview ESO 3.3 software, the normal LES resting pressure is between 13 and 43 mm Hg. Both the groups had pressures that fell within the normal range.

Furthermore, there was no statistically significant difference in LES residual pressure between opioid users and non-opioid users (Figure 2d).

Finally, in addition to analyzing the distribution of dysmotilities and sphincter pressures between the chronic opioid users and non-opioid users, we also assessed the distribution of various indications for manometry. Recall that the indication group comprised the following: Pulmonary Symptoms (PUL), Chest Pain (CP), Known Achalasia (ACH), GERD (GER), Dysphagia (DYS), and Post-op Dysphagia (POD).

**Figure 3:**
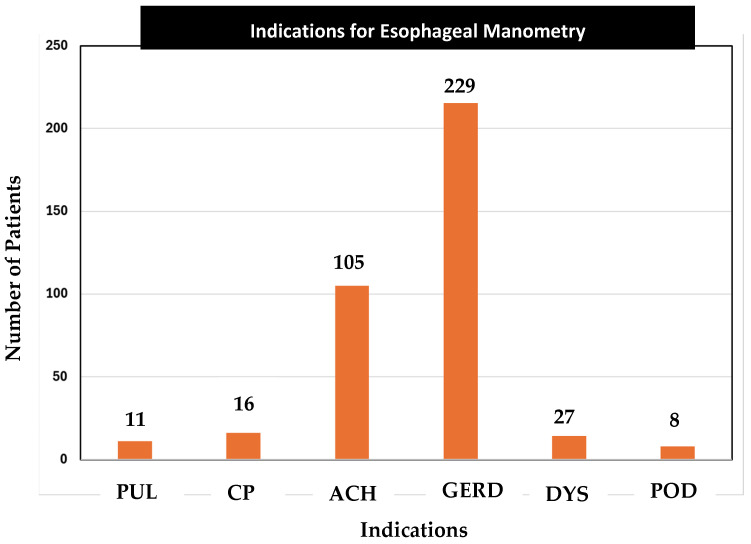
Distribution of indications for manometry.

The most prevalent indication was dysphagia (Figure 3).

**Figure 4:**
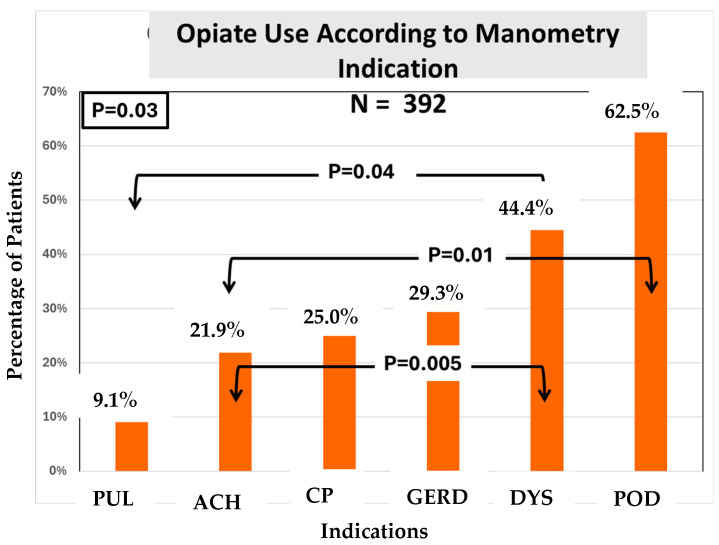
Opioid use in each indication group; *p* = 0.03.

We found that the prevalence of opiate usage was statistically different among the indications (Figure 4).

## 4. Discussion

The objectives of the study were three-fold:

To determine whether the prevalence of certain esophageal dysmotilities like Achalasia were higher in opioid users;

To examine the effect of chronic opioid therapy on the sphincter and motor pressures of the esophagus, specifically the upper esophageal sphincter;

To examine whether the indication for an esophageal manometry was related to opioid use.

Regarding the first objective—we had hypothesized that those on chronic opioid therapy would have a higher incidence of underlying esophageal dysmotilities. Recent studies have suggested a relationship between opioid use and esophageal motor abnormalities such as EGJOO and Achalasia [1,2,3,4,5]. We believed that our study pool would be a great option for this; as the literature confirms, there tends to be a greater prevalence of chronic opioid use among veterans compared to the general population [10,11]. Confirming the findings of other studies, our data supported this hypothesis. We found that veteran patients diagnosed with Achalasia had a greater proportion of chronic opioid use compared to those diagnosed with Non-Achalasia findings (UES hypertension, EGJOO, DES. IEM, Jackhammer–Nutcracker Esophagus, LES hypotension, and Normal findings (Figure 1, ***p*** = **0.001**)). However, while the previous studies noted a statistically high prevalence of opioid users with EGJOO diagnoses, our study did not observe this (*p* = 0.07).

Regarding the second objective, we assessed the mean pressure values for UES resting and residual, LES resting and residual, and DCI among opioid users and non-opioid users. Notably, our study is the first to look at this data among chronic opioid users in the veteran patient population over a 10-year period. The initial analysis showed that the average UES resting pressure in both opioid and non-opioid pressures were within normal range. Despite this, we found a statistically significant 18 mm Hg difference in mean resting UES pressure between the two groups (Figure 2a, ***p*** = **0.0001**). The mean UES resting pressures were 64.7 mm Hg and 82.7 mm Hg for opioid users and non-opioid users, respectively. We learned that veterans on chronic opioid therapy had lower average UES resting pressures compared to those not on chronic opioid therapy. Importantly, while both pressure averages remained in normal range, the opioid-induced difference should heighten awareness among all clinicians of possible opioid effects on the esophagus in addition to the stomach and bowel. Further studies are needed to investigate dose-dependent effects. While we do not have a clear causation for these effects, prior animal research can clue us into a mechanism of the effects of mu and kappa receptors on smooth muscle sphincter function [7,8]. Unlike the UES resting pressure, the mean UES residual pressures between opioid users and non-opioid users were found to be statistically similar. In other words, chronic opioid use seemed to have caused relaxation in the UES resting pressure but not in the UES residual pressures. Given the retrospective nature of the study, our current understanding of the true clinical impact is limited until future prospective studies are conducted.

Similarly, our sphincter pressure values of both resting and residual LES pressures and DCI had no statistically significant differences among opioid usage and non-opioid usage in their respective pressure values (Figure 2b, 2c, 2d, respectively). As for why the UES resting pressure was affected, there may be other factors at play such as comorbidities, concurrent medications, varying doses, opioid type, and other extrinsic factors contributing to pharyngeal muscle pressures. These variables are limitations to the retrospective nature of this database study. Our veterans are unique in that they are exposed to toxins like Agent Orange, burn pits, and warfare-related fumes. The impact of these toxins on upper esophageal sphincter dynamics remains unexplored. The UES, in close proximity to the airway, may be affected by these war-related toxins which deserve further study.

Regarding the third and last objective—whether the indication was related to the use of opiates—we found a statistically significant difference in opioid use among the indication groups (Figure 4). Here, opioid use was highest among patients presenting for manometry with an indication of post-op dysphagia. Patients with a prior diagnosis of Achalasia as an indication for manometry were found to be the second highest in opioid use. The most common indication for requesting manometry was dysphagia, and ~70% of dysphagia patients were non-opioid users despite a high frequency of opioid use among veterans in general.

In summary, the findings of our study raise further awareness of potential secondary esophageal effects in chronic opioid users, particularly in the U.S. veteran population. With the high prevalence of opioid prescriptions, clinicians must consider the impact on esophageal function.

## 5. Conclusions

Our retrospective analysis of 395 veterans over a 10-year period demonstrated the following:

Chronic opioid use was more common among Achalasia compared to Non-Achalasia dysmotilities similar to prior reports.

Chronic opioid users demonstrate lower average UES resting pressures compared to their non-opioid-using counterparts. This is the first study to find this association. However, overall UES dynamics and overall UES pressures remain within normal range in both groups.

### Limitations and Future Research Direction

In summary, it is challenging to reach conclusions on the true role of opioid therapy in esophageal dysmotility development. For instance, polypharmacy in our geriatric veteran population may be a confounding factor that limits a clear cause-and-effect relationship between the use of opioids and esophageal dysmotilities. Another limitation of our study being retrospective is that the true consumption and true effective dose of the opioid therapy are unclear. Therefore, possible dose-dependent effects cannot be excluded. As a start, prospective studies assessing dose-dependent affects of opioids on Achalasia severity and outcomes may provide a clearer explanation of the relationship.

Additionally, future long-term studies with a specific focus on UES resting and residual pressures are needed to better delineate our understanding of the relationship between opiates and the upper esophageal sphincter pathophysiology to better guide novel therapies in this challenging patient population.

Given the limitations of a retrospective database study, we suggest the best way to study any possible correlation further is to conduct a prospective study with a large sample size to assess esophageal dysmotilities and sphincter pressures following administration of opioid therapy in a dose-dependent manner. Additionally, we should assess the findings of administration of opioids on those already diagnosed with esophageal dysmotility to assess whether the symptoms are exacerbated further. Finally, to understand the clinical impact of these newfound UES muscle dynamics, future prospective studies are needed to evaluate the role of opiate use and therapeutic outcomes post intervention on each sphincter among patients with esophageal motility disorders.

## Figures and Tables

**Table 1:** Demographics of veterans presenting for manometry (*n* = 395).

*Demographic*	*Opioid* (*Yes*)	*Opioid* (*No*)	*p*-*Values*
Mean Age at Manometry (*p* = 0.48)	62 years of age	63 years of age	(*p* = 0.48)
Median Age	63 years of age	66 years of age	
Age Range	31–89	25–96	
Gender			(*p* = 0.39)
Male Gender %	87.14%	87.69%	
Female Gender %	12.86%	12.31%	
Race			(*p* = 0.22)
White	73.9%	65.1%	
Black	19.3%	26.4%	
Pacific Islander	1.8%	0.7%	
Asian	0%	1%	
Declined to Answer	4.5%	6.7%	

## Data Availability

Data is contained within the article.
